# Evaluation of an intervention mapping framework guided micronutrient literacy program for school children in Mumbai: A quasi-experimental study

**DOI:** 10.1038/s41598-026-46102-y

**Published:** 2026-04-04

**Authors:** Panchali Moitra, Aashna Mehta, Farzeen Bhatkar

**Affiliations:** Department of Postgraduate Programs and Research, Sir Vithaldas Thackersey College of Home Science (Empowered Autonomous Status), SNDT Women’s University, Santacruz West, Mumbai, Maharashtra 400049 India

**Keywords:** Children and adolescents, India, Intervention mapping, Knowledge, Attitude and practices, Micronutrient literacy intervention, Micronutrient rich foods, Nutrition education program, School nutrition intervention, Health care, Health humanities

## Abstract

This quasi experimental, pre post study evaluated the effectiveness of a school-based nutrition education intervention, developed using the Intervention Mapping (IM) framework, in improving micronutrient literacy and dietary practices among children aged 10–15 years in selected government schools in Mumbai, India. The IM steps of needs assessment, behavioral outcome mapping, theory-based intervention, educational module development, and evaluation guided the design and implementation of the 10-week program. Storytelling, peer led learning, practical demonstrations, and environmental restructuring strategies were integrated. A total of 509 children (57.2% boys; mean age ≈ 13.2 years) completed all assessments. The intervention resulted in improvements across all measured knowledge items. The perceived benefits of having fruits and vegetables and iron rich foods and willingness to learn about healthy eating and trying to eat a variety of foods improved. A significant increase in the frequency of intakes of fruits and vegetables and decrease in the consumption of fried foods, sweetened beverages, and fast foods was noted post-intervention. The IM framework strengthened program effectiveness, contributing to meaningful improvements across knowledge, attitude and practice domains. A targeted focus on micronutrient literacy in school nutrition education is essential to support existing policies and reduce micronutrient malnutrition in children.

## Introduction

Micronutrient deficiencies in school-aged children remain a critical public health challenge in India^1–3^. Recent estimates indicate that over half of Indian children have inadequate intakes of iron, vitamin A, zinc, and calcium^1,4–6^, attributed primarily to poor dietary diversity, preference for processed foods, and low awareness of micronutrient-rich foods^7–9^. In children, inadequate intakes of essential micronutrients are linked to fatigue, impaired learning, stunted growth, and weakened immunity. These persistent nutritional gaps can result in adverse long-term implications for physical growth, cognitive development, academic performance, and future productivity^2,10,11^. While improving micronutrient status in schoolchildren requires a multi-pronged approach, school-based nutrition education offers a promising strategy to build awareness and foster healthier eating habits in children^12,13^. However, for such interventions to be impactful and sustainable, they must be tailored to the specific needs, perceptions, and socio-cultural contexts of the target population.

Several studies have evaluated the impact of nutrition education programs on knowledge, attitudes and dietary practices in schoolchildren in India^9,14,15^. However, a majority of these interventions have typically relied on didactic approaches and dissemination of factual information, rather than employing comprehensive and rigorous frameworks that are grounded in behavior change theories or targeted at context-specific determinants of knowledge, attitudes, self-efficacy, and practices, leading to modest or transient changes in dietary behaviors. In addition, earlier interventions have often focused broadly on improving awareness regarding healthy eating habits^15–17^ or obesity prevention^18,19^, with limited emphasis on micronutrient-specific literacy—an area of particular concern in school children, where hidden hunger persists despite dietary sufficiency in calories^1,20^. Our study aimed to address the research gap by employing a structured, theory-driven methodology, the Intervention Mapping (IM) approach, to develop a school-based nutrition education program focused on improving micronutrient-related awareness, attitudes, and adequacy in children.

Micronutrient literacy relates to an individual’s ability to acquire, process and apply the information about micronutrients (vitamins and minerals) to make informed dietary choices. By contrast, general nutrition education or food literacy tends to emphasize broader concepts like balanced meals, portion size, label reading or food preparation and purchasing behaviors. Despite improvements in food availability and energy intake, micronutrient deficiencies continue to persist among school-aged children, leading to suboptimal growth, impaired immunity, reduced cognitive performance, and poor academic outcomes. Micronutrient malnutrition often remains unrecognized as it is not accompanied by overt signs of undernutrition or energy deficiency and therefore may not be adequately addressed through general nutrition education that focuses primarily on balanced diets, food groups, or caloric adequacy. In this context, interventions that move beyond broad dietary messages and instead target micronutrient-specific knowledge, attitudes, and dietary practices (KAP) are increasingly warranted. A concerted, targeted effort to improve knowledge regarding functions and sources of essential vitamins and minerals, and perceptions and skills required to select and consume micronutrient rich foods would allow individuals to recognize signs of hidden hunger in seemingly adequate diets and tailor diets to prevent nutrient deficiencies and their long-term health consequences. In our study, the micronutrient literacy was operationalized as a multidimensional construct encompassing children’s knowledge of key vitamins and minerals, attitudes towards the importance and relevance of micronutrients for health, and their dietary practices related to intentional consumption of micronutrient-rich foods. The primary outcomes of interest were changes in micronutrient-specific KAP and the frequency of consumption of micronutrient-rich food groups, reflecting both literacy gains and behavioral translation.

Previous studies have used different theoretical frameworks, such as the Health Belief Model, the Theory of Planned Behavior, or the Social Cognitive Theory, to guide the development and implementation of nutrition education interventions for children^16,21–23^. While these behaviors change theories offer valuable insights into individual-level motivation and behavior^24^, the IM’s step-by-step framework—from needs assessment to defining performance objectives, selection of appropriate theory-based methods, and development of implementation and evaluation strategies ensures a culturally relevant, and focused approach to address the barriers and facilitators of intended outcomes in the target population^25,26^. Moreover, IM emphasizes the use of interactive and engaging methods for enhancing participant engagement and retention and stakeholder inputs throughout the process, resulting in interventions that are not only more effective but also more sustainable and scalable compared to conventional approaches^27^. Given the multifactorial nature of micronutrient malnutrition and the complex behaviors involved in improving dietary practices in children and adolescents, we believe that the IM approach would offer a comprehensive and rigorous framework to design and implement a nutrition education program that is theoretically sound, evidence-based, and locally relevant.

The steps of the IM protocol have been extensively used in studies conducted across the world. A school-based program, Healthy Lifestyles Programme (HeLP) in the UK, was developed using the first four steps of the IM protocol to prevent obesity in children aged 9–10 years by promoting healthier eating habits and reducing sedentary behavior^28^. Another school-based intervention aimed at increasing fruit and vegetable consumption among primary school children used IM for a systematic development of a multi-component, age-specific curriculum involving classroom education and parental participation in the Netherlands^29^. A comprehensive intervention was designed to improve food and nutrition literacy among Iranian Kurdish primary school children. The 16-week program combined educational sessions for students, parents, and school staff, employing various teaching strategies such as lectures, group discussions, and practical activities^27^. A multi-centre European project, The IDEFICS (Identification and prevention of Dietary- and lifestyle-induced health EFfects In Children and infantS) project, applied the IM protocol to develop a community-based intervention for preventing childhood obesity across multiple European countries. The intervention included school-based curriculum activities, environmental changes, and parental involvement^30^. These studies demonstrate the versatility and effectiveness of the IM approach in designing tailored, theory-based nutrition education programs that address specific behavioral determinants and are adaptable to various cultural and contextual settings.

With regards to micronutrient-related awareness, perceptions, and dietary practices, several studies from India and other countries have highlighted poor knowledge related to healthy eating behaviors, dietary intake recommendations, and consequences of deficiencies on health in children and adolescents. In a study conducted among adolescent girls in Hyderabad, only 25% had good knowledge about anemia^31^. A study in Orissa reported that over 80% of respondents lacked knowledge about iodine deficiency disorders^32^ and a study in children aged 2–16 years in Maharashtra found that the daily calcium intake was only 53% of the recommended dietary allowance with the majority of children having cereal-pulse-based dietary patterns, and limited consumption of milk and milk products^33^. Evidence from other low and low middle-income countries suggests similar findings. For instance, a study in Bangladesh observed that 75.4% of adolescent girls had not heard of anemia, 84.8% did not know how to prevent anemia, and 69.6% were unaware of iron-rich foods, indicating a significant knowledge gap^34^. In Ethiopia, research among early adolescents showed that only 31.4% knew the food sources of iron, and a mere 4.4% were able to identify animal-based foods as rich sources, highlighting misconceptions about iron-rich diets^35^.

Specific studies evaluating the knowledge and dietary practices related to zinc and calcium deficiency among Indian children are limited. However, there is enough data to suggest gross inadequacies in zinc and calcium intakes in children in India in both rural and urban settings, particularly in children belonging to low-income households due to limited dietary diversity, low purchasing power, poor food environments, and lack of awareness related to healthy eating behaviors and nutrition^1,36^. These findings underscore the importance of nutrition education programs that address specific micronutrient deficiencies, correct misconceptions, and promote adequate dietary practices among children and adolescents. There exists a need for structured, multimodal nutrition education interventions that target the consumption of micronutrient-rich foods, particularly among vulnerable populations such as children and adolescents in urban low-income settings. Moreover, given the consistent prevalence of undernutrition^1,37^and the increasing burden of overweight and obesity in school children^38–40^, even among those who belong to socioeconomically disadvantaged households^41^, it is imperative to design interventions that emphasize micronutrient-rich food intake within the context of existing resources, cultural preferences, and locally available and affordable healthy options.

Therefore, we conducted this study to evaluate the effectiveness of a school-based nutrition education intervention—designed using the Intervention Mapping approach—in improving knowledge, attitudes, and practices related to the intake of micronutrient-rich foods among 10–15-year-old children in selected government aided schools in Mumbai. The specific objectives were (1) to assess baseline knowledge, attitudes, and practices (KAP) related to micronutrient-rich food intake, (2) to design a theory-informed, participatory nutrition education curriculum focused on key micronutrients (iron, vitamin A, calcium and zinc) using the IM approach, (3) to deliver the education sessions over the 10-week trial duration using interactive, age-appropriate resources at schools, and (4) to assess changes in KAP and self-reported intake of micronutrient-rich foods from pre- to post-intervention. We hypothesize that strengthening micronutrient literacy among school children may offer a focused and contextually relevant strategy to address hidden hunger by enabling informed food choices that support micronutrient adequacy even within energy-adequate diets.

## Methods

### Study design and setting

The intervention was conducted across three government-aided educational institutions located in the selected western and central suburbs of Mumbai. Schools were purposively selected based on the following criteria: (1) being government-run or government-aided and having co-educational enrollment, (2) the willingness of school authorities to participate in the intervention program, and (3) attended by students primarily from low- to middle-income families. Formal permissions were obtained from the school principals before the start of the data collection.

### Participant sampling

Within each selected school, students aged 10–15 years, attending grades 5 to 9, were targeted. To ensure a representative sample, we randomly selected two to three sections per grade, in collaboration with school staff, as the sampling frame. All students in these selected classes were invited to participate in the study. A total of 588 students were enrolled after obtaining written informed consent from the school principals and written assent from the students, in accordance with ethical research protocols. Of these participants, 509 children (291 boys and 217 girls) completed both baseline and endline KAP assessments and were included in the data analysis. The intervention was conducted over three months, from January to March 2024, with sessions delivered during regular school hours in coordination with class teachers and the school supervisors.

### Sample size

Sample size estimation was performed using Epi Info version 3.03a (Centers for Disease Control and Prevention, Atlanta, USA). Assumptions were informed by prior Indian school-based nutrition education studies reporting baseline knowledge levels of approximately 35–45%^17,22^and average post-intervention improvements of 10–20%^9,42,43^. Using a paired pre–post design (McNemar test) with a 5% significance level, 80% power, and an expected 15% absolute improvement in composite KAP scores, the required sample was estimated for a design effect of 2.0 (assuming an intra-class correlation of 0.02 and an average class size of 50) and a 20% allowance for attrition. This resulted in a final recruitment target of 528 school children (220 per age stratum, 10–12 years and 13–15 years), providing sufficient statistical power to detect meaningful changes both in the overall sample and within each age group.

### Steps in the development of the intervention program model

The intervention was developed and implemented across the six steps of the IM framework, comprising formative research, identification of behavior change objectives, selection of a theoretical framework for program design followed by implementation and evaluation^25^.

#### Need assessment

The first step involved a comprehensive needs assessment to identify the knowledge gaps and the key behavioral and environmental determinants of the intake of micronutrient-rich foods among school-aged children. This included a literature review of the prevalence of iron, vitamin A, and calcium deficiencies in the region, as reported in national nutrition surveys and local public health databases to establish the need of the intervention, followed by a scoping review of existing school-based nutrition education programs and a thorough desk review of the existing nutrition related content in the academic textbooks of grades 5–9 of the Maharashtra state board of education. To further understand the existing perceptions, awareness regarding micronutrients, nutrition, and in general, the healthy eating habits, and streamline the integration of intervention into the academic calendar for the semester, we conducted in-depth interviews with the principals (n = 2) and selected teachers (n = 5) of the participating schools.

#### Development of matrices of behavior change

Based on insights gathered during the needs assessment, the second step involved the development of the matrices of change objectives. This step helped to define desired behavioral outcomes and related changes across micronutrient-related knowledge, attitude, and practice domains. The behavior change outcomes included (a) improvement in children’s knowledge scores (b) shift in composite positive attitude scores in the KAP questionnaire (c) improvement in dietary practice scores, measured in terms of the frequencies of intakes of healthy and unhealthy foods and (d) increase in the self-reported frequency of consumption of micronutrient rich food groups such as whole grains, fruits, vegetables and animal source foods by children, estimated using a qualitative food frequency questionnaire, developed by the researchers based on existing questionnaires used in India^32,33,44,45^.

#### Selection of a theoretical framework

The third step, as per the IM approach, was the selection of a theoretical framework to guide the intervention. Our intervention program was grounded in the Social Cognitive Theory, a well-documented and extensively used framework in intervention programs that emphasizes behavior change through observational learning, reinforcement, and self-efficacy^24^. This theory was selected for its strong applicability to school-based health promotion based on a literature review of similar education programs^46,47^. The key strategies that were included in our program design were modeling through storytelling and role-play featuring relatable peer role models, guided practice through classroom demonstrations, such as healthy snack preparation, reinforcement via a reward system for bringing fruits or vegetables in tiffin boxes, and environmental restructuring using age-appropriate posters and visual prompts placed in classrooms and shared spaces to reinforce key messages.

#### Program design and implementation protocol

The next step was program design and implementation planning. The intervention was deigned to span ten weeks, integrated into the academic calendar of the participating grades, with one 30-min session conducted each week per class. The curriculum comprised age-appropriate modules on food groups and dietary diversity, the role of micronutrients (particularly iron, vitamin A, and calcium), signs and symptoms of anemia and other micronutrient deficiencies, common dietary habits related misconceptions (related to taste, convenience, and affordability of healthy foods) and strategies to prepare healthy tiffin (lunchbox) meals. Educational materials such as posters and flipcharts, worksheets, short videos, and take-home activities were developed. All modules were reviewed and validated by a panel of three subject-matter experts in public health and nutrition to ensure scientific accuracy, cultural appropriateness, and age-relevance.

#### Implementation of intervention

The next steps involved program implementation in schools and evaluation of the effectiveness of the developed model in improving intended behavior change outcomes. To facilitate implementation, permissions were obtained from school principals, and planning meetings were held with teachers to integrate sessions into the academic schedule. A toolkit consisting of educational materials was shared with the school staff prior to the program rollout for review and feedback.

The intervention was implemented using an interactive approach designed to optimize student engagement. Educational sessions were delivered in English, Hindi, and Marathi to ensure language accessibility. Colorful posters and visual aids illustrated key topics such as the functions of iron, symptoms of anemia, and sources of vitamin C. A mnemonic device, “Signal Foods”, was introduced to help children remember iron-rich foods. Interactive activities, including storytelling, games, and role-play, were facilitated by trained educators and student volunteers to promote active learning. Students also engaged in a hands-on card activity in which they matched micronutrients to their functions, food sources, and deficiency diseases using a templated chart. Concepts such as the food pyramid and the “My Plate” model were introduced to provide practical guidance on balanced eating. These strategies were aimed at enhancing both knowledge retention and the development of healthy eating behaviors.

#### Program evaluation

Finally, the evaluation of the intervention was carried out through both process and impact assessments. For process evaluation, qualitative feedback was collected from students and teachers, post-intervention, to assess relevance, feasibility, and student engagement. The impact evaluation employed a pre-post design, using a structured KAP questionnaire and a food frequency questionnaire (FFQ) to assess changes in dietary behaviors related to the intake of micronutrient-rich foods. The primary outcomes were changes in composite KAP scores and frequency of consumption of micronutrient-rich food groups pre to post intervention. The secondary outcomes included the percentage of students correctly identifying micronutrient-rich foods, and mean changes in attitude scores toward healthy eating, and weekly consumption of healthy and unhealthy foods.

### Data collection and variables

The data was collected using an interviewer-designed, self-reported KAP questionnaire adapted from the Anemia Mukt Bharat tool kit, a comprehensive resource developed by the Ministry of Health and Family Welfare, Government of India for addressing anemia and micronutrient deficiencies in India. To ensure content validity, the questionnaire was reviewed for clarity, relevance, and comprehension by three experts in the field of public health and nutrition. The reviewers felt a need to translate the English language questionnaire into local languages- Hindi and Marathi to improve linguistic accessibility and accurate interpretations by the participants. Hence, we translated the questionnaire and then back-translated it to English to ensure comprehension and response accuracy, thereby enhancing the reliability and validity of the data collected.

Overall, the questionnaire comprised 56 questions, divided into four sections: socio-demographic details, knowledge, attitudes, and practices related to frequency of consumption of micronutrient rich foods such as fruits, green leafy vegetables, milk and milk products, nuts and seeds and others. The socio-demographic section included three questions related to the participants’ age, gender, and family size. The knowledge section consisted of 21 questions designed to assess participants’ understanding of the functions, sources, and signs and symptoms of deficiency diseases associated with iron, calcium, vitamin A, and other micronutrients, including vitamin C, vitamin D, iodine, and zinc. These items targeted the assessment of knowledge about micronutrients and their functions, the concept of hidden hunger, signs and symptoms of inadequate intakes, dietary sources, negative consequences of deficiencies, and food-based micronutrient deficiency prevention strategies such as dietary diversification and food fortification. Each correct response was given 1 and incorrect/ don’t know options as 0, with total scores ranging from 0 to 21.

The 10-item attitudes section assessed participants’ perceptions related to the importance of consumption of micronutrient-rich foods, recognition of anemia as a serious health problem, and perceived barriers and facilitators of healthy eating behaviors on a five-point Likert scale ranging from “strongly agree” to “strongly disagree”, scored from 0 to 4. The practice section was developed to understand the frequency of consumption of 22 food items across the seven food groups of (1) cereals, (2) pulses and legumes, (3) milk and milk products, (4) nuts and oilseeds, (5) vegetables, (6) fruits and (7) eggs, meat and fish. A 30-day recall period was used for the FFQ as it represents a feasible and developmentally appropriate timeframe for children, allowing assessment of habitual intakes while minimizing recall burden and improving response accuracy. The specific food items within each food group were selected based on the commonly consumed foods by children as reported in previous studies^48,49^. An additional 8 food items, under three food categories (fried foods, sugary drinks and snacks, and fast foods) assessed the frequency of consumption of unhealthy foods by children. Participants were asked to indicate how often they had consumed each food item during the past 30 days, using the frequency options- 2–3 times per day, every day, 2–3 times per week, 2–3 times per month, and rarely/never (scored from 4 to 0, respectively). To calculate the cumulative frequency score for each food group, the scores for individual food items within that group were summed.

### Data analysis

Data were entered and analyzed using IBM SPSS Statistics version 26.0 (IBM Corp., Armonk, NY, USA). Descriptive statistics were used to summarize sociodemographic characteristics and baseline measures of knowledge, attitudes, and practices (KAP), as well as dietary intake frequencies. Categorical variables were presented as frequencies and percentages, while continuous variables were reported as means and standard deviations (SD). To assess the effectiveness of the intervention, pre- and post-intervention scores for KAP and food frequency data were compared. For normally distributed continuous variables, paired-sample t-tests were conducted to evaluate mean differences in knowledge, attitude, and practice scores, as well as the frequency of consumption of healthy and unhealthy foods before and after the intervention. For non-normally distributed data, the Wilcoxon signed-rank test was used. The McNemar test was employed to evaluate changes in categorical variables such as the proportion of students correctly identifying specific micronutrient-rich foods. A significance level of *p* < 0.05 was considered statistically significant. All analyses were performed on an intention-to-treat basis, including all students with both pre- and post-intervention data available. Missing outcome data were not imputed, as the proportion of missing data was low and largely attributable to absence on the day of follow-up assessment. To further explore the relationship between key covariates (e.g., age, gender, grade level) and improvements in outcome scores, multivariate linear regression models were employed. These models helped determine the predictors of change in KAP scores and dietary behaviors.

## Results

The mean age of the participants (n = 509, 57.2% boys) was 13.8 (1.1) years. Most participants (85.5%) belonged to families with more than three members, 39.7% were between 10 and 12 years and 46.8% attended grades 8–9 (Table 1)

**Table 1:** Demographic characteristics of participants (n = 509).

Variables	Frequency, n (%)
*Sex*	
Boys	291 (57.2)
Girls	217 (42.8)
*Age (in years)*	
10–12 13–15	202 (39.7) 307 (60.3)
*Classes attended*	
Grades 5–7	271 (53.2)
Grades 8–9	238 (46.8)
*Family size*	
< 3	74 (14.5)
> 3	435 (85.5)

^†^Data are presented as numbers and percentages.

### Pre to post intervention changes in knowledge and attitude scores

Table 2 shows significant improvements in the proportion of children providing correct responses to all knowledge questions after the intervention. The knowledge items related to the function and sources of micronutrients, such as foods aiding iron absorption, role of zinc in body, and vitamin C deficiency showed substantial improvements, ranging from 137 to 350%. Improvements (60–100%) were noted for vitamins A, D, iodine, and anemia-related concepts, while smaller but significant changes (~ 20–50%) were observed for topics related to specific nutrients and their food sources. Awareness regarding anemia and its symptoms, consequences and prevention strategies, the functions of vitamins A, D, and iodine and food fortification also showed significant improvements in post intervention assessments (all *p* < 0.001).

**Table 2:** Pre to post-intervention changes in proportion of children providing correct responses to knowledge items in the study (n = 509).

Question	Pretest	Post-test	Percent change (%)	*P* value
What are nutrients	172 (28.0)	252 (41.0)	46.4	< 0.001***
What are micronutrients	121 (19.7)	285 (46.4)	135.5	< 0.001***
Function of iron	159 (25.9)	286 (46.6)	79.9	< 0.001***
Dietary sources of iron	222 (36.2)	290 (47.2)	30.4	< 0.001***
Foods that help in iron absorption	52 (8.5)	236 (38.4)	352.9	< 0.001***
Function of calcium	238 (38.8)	293 (47.7)	22.9	< 0.001***
Dietary sources of calcium	188 (30.6)	288 (46.9)	53.3	< 0.001***
Functions of iodine in body	124 (20.2)	273 (44.5)	120.3	< 0.001***
Functions of vitamin A in body	146 (23.8)	293 (47.7)	100.4	< 0.001***
Dietary sources of vitamin A	250 (40.7)	300 (48.9)	20.1	< 0.001***
Role of vitamin C in the body	169 (27.5)	278 (45.3)	64.7	< 0.001***
Role of zinc in growth and immunity	76 (12.4)	239 (38.9)	213.7	< 0.001***
Symptoms of vitamin D and calcium deficiency in children	159 (25.9)	273 (44.5)	71.8	< 0.001***
Iodine deficiency diseases	144 (23.5)	286 (46.6)	98.3	< 0.001***
Signs of vitamin A Deficiency	153 (24.9)	279 (45.4)	82.3	< 0.001***
Signs of Vitamin C deficiency	114 (18.6)	270 (44)	136.6	< 0.001***
What is anemia	173 (28.2)	289 (47.1)	67.0	< 0.001***
Symptoms of anemia	191 (31.1)	286 (46.6)	49.8	< 0.001***
Consequences of anemia for children	149 (24.3)	228 (37.1)	52.7	< 0.001***
Prevention strategies for anemia	180 (29.3)	294 (47.9)	63.5	< 0.001***
Concept of food fortification	143 (23.3)	269 (43.8)	87.9	< 0.001***

*p < 0.05, ** *p* < 0.01, ***p < 0.001.

Analyses of attitude item scores showed significant improvements in perceived benefits of having fruits and vegetables (3.21 (1.2) vs 3.77 (1.2), *p* < 0.001), and iron rich foods for improved energy (3.08 (1.2) V 3.92 (1.1), *p* < 0.001) and motivation and willingness to eat healthy foods, such as wanting to learn more about healthy eating (3.57 (1.1) vs 4.14 (0.8), *p* < 0.001) and trying to eat a variety of foods to get the required micronutrients in diet ( 2.38 (1.1) vs 3.31 (1.3), *p* < 0.001) (Table 3). The results also indicated a positive shift in perceptions about anemia as a serious health problem, eating rice and wheat being not enough to meet the body’s nutrient needs and adverse consequences of skipping meals or eating too much of sugary drinks and fast foods on health.

**Table 3:** Pre to post-intervention changes in attitude item scores among participants (n = 509)

Attitude related items^#^	Pre	Post	*p* value
Eating fruits and vegetables every day is important for staying healthy and strong	3.21 (1.2)	3.77 (1.2)	< 0.001
Iron-rich foods like leafy greens and whole grains help prevent tiredness and weakness	3.08 (1.2)	3.69 (1.1)	< 0.001
Skipping meals or eating too much of sugary drinks and fast foods can make me fall sick more often	3.08 (1.3)	3.65 (1.2)	< 0.001
Eating only rice or chapati does not give my body all the nutrients it needs	3.07 (1.3)	3.47 (1.2)	< 0.001
Even if I don’t feel sick, I might still have low levels of nutrients in my body	2.69 (1.2)	3.21 (1.3)	< 0.001
Anemia is a serious health problem that can affect how well I do in school and sports	3.41 (1.1)	4.01 (1.0)	< 0.001
It is hard to eat healthy foods because they are not always available at home or school	3.33 (1.1)	3.22 (1.1)	0.065
I don’t eat fruits and vegetables as I don’t like the taste	3.18 (1.1)	3.20 (1.2)	0.231
I want to learn more about healthy foods that can make my body and brain work better	3.57 (1.1)	4.14 (0.8)	< 0.001
I will try eating a variety of healthy foods to get all the vitamins and minerals I need	2.38 (1.1)	3.31 (1.3)	< 0.001

Values are provided as mean (standard deviation).

^#^Attitude items were scored on a five-point Likert scale ranging from “strongly agree” to “strongly disagree”, scored from 0 to 4.

### Pre to post intervention changes in dietary practice scores

Table 4 presents pre to post intervention changes in the frequency of consumption of healthy foods across seven food groups and also of unhealthy food items such as sugary drinks and snacks, and fried and fast foods. A significant increase was noted in the intake of fruits (*p* < 0.001), vegetables (*p* < 0.001), and animal-source foods such as eggs, fish, and poultry (*p* = 0.041). Conversely, the consumption of fried foods (*p* = 0.016), sugary snacks and beverages (*p* < 0.001), and fast foods (*p* < 0.001) significantly decreased post-intervention. No significant differences were observed in consumption of cereals, pulses, milk products, or nuts and seeds.

**Table 4:** Pre to post-intervention changes in frequency of consumption of specific food groups among participants (n = 509)

Food groups	Pre intervention	Post intervention	*p* value
Cereals (4 items- rice, wheat, millets and white bread/pav)	12.83 (4.9)	13.23 (5.64)	0.09
Pulses and legumes (2 items- lentils and beans such as chickpea/ kidney beans)	3.47 (1.9)	3.24 (1.3)	0.541
Milk and milk products (3 items – Milk, Curd/ yoghurt, cottage cheese)	4.86 (1.6)	4.52 (1.2)	0.134
Fruits (4 items -Vitamin A rich fruits (mango/papaya/melons), citrus fruits, banana, apple/ other fruits)	5.97 (2.83)	8.84 (2.64)	< 0.001***
Vegetables (4 items- green leafy vegetables, beetroot/carrots/ tomato, capsicum/cluster beans/pumpkin and other vegetables	5.60 (1.50)	6.86 (1.43)	< 0.001***
Nuts and seeds (2 items – nuts and seeds)	1.40 (0.9)	1.37 (0.8)	0.341
Eggs, fish and poultry/meats (3 items)	2.76 (1.9)	3.04 (1.9)	0.041*
Fried Foods (3 items – samosa/vada, bhajiya, puri/fafda/namkeen)	5.39 (1.1)	5.13 (1.8)	0.016*
Sugary drinks and snacks (2 items- sweetened beverages/ carbonated beverages, cakes/icecream/desserts)	3.72 (1.76)	2.76 (1.0)	< 0.001***
Fast food (3 items- noodles/fried rice/ Manchurian, sevpuri/panipuri, pav bhaji/frankie)	4.96 (1.7)	4.15 (1.9)	< 0.001***

^#^Frequency of consumption of each food item were reported as 2–3 times/day, every day, 2–3 times a week, 2–3 times a month, and rarely/never (scored 4 to 0).

^##^Values provided in the table are mean (standard deviation) of cumulative scores calculated for different food items within a specific food group.

Paired sample t tests were performed to calculate the *p* values.

### Comparison of changes in KAP scores between 10–12 year and 13–15-year-old children

Within group comparisons showed significant post intervention improvements in knowledge, attitude, and practice scores among both age groups (10–12 years and 13–15 years). However, older children (13–15 years) demonstrated significantly higher post-intervention gains in knowledge and attitude (*p* < 0.001) (Table 5). Improvements in healthy food consumption were significant in both groups, with no inter-group difference (*p* = 0.268). Unhealthy food consumption declined significantly in both groups (*p* < 0.001), indicating positive behavioural changes across ages.

**Table 5:** Comparison of Pre to post-intervention changes in knowledge, attitude and practice scores between school children aged 10–12 years (n = 202) and 13–15 years (n = 307).

Variables	Survey period	10–12 years (n = 202) Mean (SD)	13–15 years (n = 307) Mean (SD)	*P* value ^b^
Knowledge	Pre intervention Post intervention *p* value (within group) ^a^	12.29 (4.61) 17.38 (6.03) < 0.001**	15.36 (5.07) 19.55 (4.18) < 0.001**	< 0.001** < 0.001**
Attitude	Pre intervention Post intervention *p* value (within group)	26.59 (7.38) 32.47 (10.05) < 0.001**	28.09 (7.21) 38.18 (8.45) < 0.001**	0.0233 < 0.001**
Dietary practice (Healthy food consumption) ^#^	Pre intervention Post intervention *p* value (within group)	34.25 (9.89) 49.13 (14.46) < 0.001**	35.05 (6.41) 44.04 (11.50) < 0.001**	0.268 < 0.001*
Dietary practice (Unhealthy food consumption) ^##^	Pre intervention Post intervention *p* value (within group)	13.44 (5.08) 11.97 (5.63) < 0.001**	16.28 (5.21) 12.18 (5.19) < 0.001**	< 0.001** 0.666

* SD* standard deviation, *p value < 0.05, **p value < 0.001.

^a^ Significance level tested using paired t test.

^b^ Significance level tested using independent sample t test.

^#^ Composite healthy food consumption scores were calculated by summing frequency scores of consumptions of 22 items across seven food groups (details in the methods section).

^##^ Composite unhealthy food consumption scores were calculated by summing frequency scores of consumptions of 8 items across three categories (fried foods, sugary drinks/snacks, and fast foods).

### Predictors of change in KAP scores

Multivariate linear regression analyses were conducted to identify predictors of change in composite KAP scores, and in the frequency of consumption of micronutrient rich healthy foods among schoolchildren (n = 509), after adjusting for age, gender, grade level, baseline scores, and intervention session attendance (Table 6). The models explained a moderate proportion of variance in outcomes, with R^2^ values ranging from 0.17 to 0.23, indicating meaningful predictive strength for behavioral data in field settings.

**Table 6:** Multivariate Linear Regression Models for Predictors of Change in KAP Scores and Frequency of Consumption of Micronutrient-Rich Healthy Foods in Children (n = 509).

Predictor variable	Δ Knowledge score (β, 95% CI)	*p* value	Δ Attitude score (β, 95% CI)	*p* value	Δ Practice score (β, 95% CI)	*p* value	Δ Frequency of micronutrient-rich foods (β, 95% CI)	*p* value
Age (years)	0.12 (0.05, 0.19)	0.002*	0.08 (0.01, 0.15)	0.025*	0.03 (– 0.04, 0.10)	0.412	0.05 (0.01, 0.09)	0.018*
Gender (Male = 1, Female = 0)	0.10 (– 0.05, 0.25)	0.191	0.22 (0.09, 0.35)	< 0.001**	0.18 (0.04, 0.32)	0.012*	0.16 (0.04, 0.29)	0.009*
Grade 6	0.05 (– 0.10, 0.20)	0.501	0.02 (– 0.13, 0.17)	0.796	0.06 (– 0.09, 0.21)	0.439	0.09 (– 0.03, 0.21)	0.146
Grade 7	0.18 (0.02, 0.34)	0.029*	0.14 (0.01, 0.27)	0.035*	0.17 (0.02, 0.32)	0.024*	0.11 (0.00, 0.22)	0.045*
Grade 8	0.25 (0.09, 0.41)	0.002*	0.21 (0.06, 0.36)	0.007*	0.22 (0.07, 0.37)	0.005*	0.20 (0.07, 0.32)	0.002*
Grade 9	0.42 (0.56, 0.28)	< 0.001**	0.37 (0.52, 0.22)	< 0.001**	– 0.40 (– 0.55, – 0.25)	< 0.001**	– 0.33 (– 0.45, – 0.21)	< 0.001**
Attendance (≥ 80% = 1)	0.30 (0.12, 0.48)	< 0.001**	0.27 (0.10, 0.44)	0.003*	0.29 (0.11, 0.47)	0.002*	0.31 (0.16, 0.46)	< 0.001**

*CI* Confidence interval, β: Unstandardized regression coefficient.

Model R^2^: Knowledge = 0.21, Attitude = 0.18, Practice = 0.17, Frequency = 0.23.

Δ indicates change in composite knowledge, attitude and practice scores from pre- to post-intervention.

All models, adjusted for age, gender, grade level, baseline score of outcomes, and session attendance.

Age was observed as a significant positive predictor of improvements in knowledge (β = 0.12, *p* = 0.002), attitude (β = 0.08, *p* = 0.025), and frequency of micronutrient-rich food consumption (β = 0.05, *p* = 0.018), but not for practice score changes. Boys showed greater post-intervention improvements in attitude (β = 0.22, *p* = 0.001), practice (β = 0.18, *p* = 0.012), and micronutrient-rich food intake (β = 0.16, *p* = 0.009) than girls, though knowledge gains were not significantly different. Being in Grade 7 or 8 was associated with significantly greater gains in all KAP domains (β range = 0.14–0.25, all *p* < 0.05) and having attendance of ≥ 80% emerged as a positive predictor of improvement in all outcomes (β = 0.27–0.31, all *p* ≤ 0.003). Interestingly, being in grade 9 showed significant associations with improvements in knowledge and attitude but were negatively associated with practice scores and consumption of healthy foods.

## Discussion

This quasi-experimental study employed the IM framework to design and implement a school-based nutrition education intervention to improve knowledge, attitudes, and practices (KAP) related to micronutrient intake among children aged 10–15 years in Mumbai, India. The intervention resulted in consistent and statistically significant improvements across all measured knowledge items, suggesting that interactive, age-appropriate modules can successfully improve nutrition and health related awareness in this age group. Besides improvements in knowledge scores, we observed positive shifts in attitude scores and desirable changes in dietary practices such as an increased consumption of fruit, vegetable and animal-source foods and reduced intake of sugary drinks, fast foods and fried foods, highlighting the effectiveness of the educational intervention in not only improving conceptual understanding of essential nutrients and diet–health relationships, but also translating into better attitudes and improved dietary behaviors among participants.

Our findings align with existing evidence that have reported well-designed school-based nutrition interventions to achieve meaningful changes in nutrition knowledge, attitudes and dietary behaviors in children and adolescents^43,47,50–52^. Systematic reviews of effective nutrition education interventions have also reported that school programs that combine classroom education with behavior-focused strategies and environmental supports are most likely to result in improvements in fruit and vegetable intake and reductions in unhealthy food consumption^53–56^. In our study, we applied the Intervention Mapping framework to align needs, context and methods and iteratively develop age-appropriate education materials. By explicitly linking determinants to behavior change methods and designing context-relevant materials, IM approach has been known to increase the likelihood that intervention components target the psychosocial mediators (knowledge, attitudes, self-efficacy, subjective norms) that underpin behavior change^25,27^. Furthermore, we used focused pedagogical strategies such as demonstrations, simple take-home messages, reminders, family assignments and practical examples to consolidate learning, which must have contributed to favorable changes in attitudinal variables and dietary practices of the participants. Substantially large percentage increases in knowledge scores can be attributed to poor knowledge regarding at functions and deficiency symptoms of specific micronutrients at baseline, further underscoring the importance of micronutrient-focused literacy initiatives in school settings. The observed reductions in unhealthy food consumption and increases in fruit/vegetable and animal-source food intake are consistent with interventions that include persuasive messaging, goal setting and environmental prompts^22,43,57^.

Although significant post-intervention improvements were noted in the intake of fruits, vegetables, and animal-source foods, we did not observe changes in the consumption of cereals, pulses, milk products, or nuts and seeds. This finding may be explained by the fact that these foods already constitute staple components of the habitual Indian diet, with limited variability in daily consumption by children. Cereal-based foods (rice, wheat) form the dietary base across most households and are perhaps less influenced by nutrition education since they are not discretionary choices but rather routine staples determined by household availability and cultural norms^58–60^. Similarly, the intake of pulses, milk, and nuts may depend more on household purchasing capacity, food accessibility, and parental control over meals than on individual child preferences or awareness. Structural and economic constraints are known to play an important moderating role in shaping dietary behavior change, particularly for relatively higher-cost and perishable foods such as fruits, nuts, and milk, which may be deprioritized in households facing budgetary constraints or competing food needs. Our study was conducted in government schools catering to households belonging to low socioeconomic backgrounds, which could have impacted the availability and affordability of these food items in the participant households. This can explain the finding that despite children demonstrating increased awareness and intent following nutrition education, the consumption of these food items showed modest gains, highlighting the need for complementary strategies that address economic access and household-level decision-making to sustain behavior change. Similar studies conducted in South Asia have also noted that even when school-based nutrition interventions improve knowledge and attitudes, economic and structural barriers can constrain behavioral translation, especially for higher-cost or less-available items such as milk or nuts^55,61^. These results reinforce that educational strategies alone may not suffice to change consumption of resource-dependent food groups without parallel improvements in food environment and affordability.

Beyond demonstrating improvements in nutrition education outcomes, this study advances the concept and measurement of micronutrient literacy by operationalizing it as a distinct, multi-dimensional construct encompassing micronutrient-specific knowledge, attitudes, and practices rather than broad dietary awareness alone. By explicitly linking literacy components to targeted dietary behaviors relevant to anemia and micronutrient deficiencies, the study moves the field beyond generic nutrition messaging toward a more targeted and measurable framework. This approach provides a practical template for assessing micronutrient literacy in school settings and strengthens its relevance for designing, implementing, and evaluating micronutrient-focused interventions in populations at risk of hidden hunger.

Another key finding of our investigation was that while older children (13–15 years) demonstrated significantly greater post-intervention gains in knowledge and attitude scores (*p* < 0.001), they did not show superior improvements in healthy or unhealthy food consumption patterns compared with younger children (10–12 years). This finding can be attributed to the older participants having greater cognitive capacity for conceptual learning, but the behavioral adoption may have been moderated by habitual eating habits, peer influences, and autonomy in food choices that emerge during mid-adolescence^62,63^. In contrast, early adolescence (10–12 years) represents a transitional stage where cognitive receptivity coexists with continued parental and school influence—providing a critical window of opportunity for instilling healthy eating behaviors before lifestyle patterns consolidate^64,65^. While further investigations would be required in broader and more diverse groups to validate these findings, our results suggest that younger adolescents are more amenable to structured educational messages and reinforcement through parental modelling and school food policies^47,66^. Age-differentiated content and delivery strategies, such as interactive, discussion-based, and peer-led components can be considered for older students (13–15 years) who may benefit from greater autonomy, social influence, and opportunities for peer engagement.Overall, these findings suggest that combining early initiation of nutrition education with supportive environmental strategies targeting food affordability and availability could maximise both knowledge translation and sustained dietary change.

Regression analyses revealed that age, gender, and attendance were consistent predictors of positive change across knowledge, attitude, and dietary behavior outcomes. Older children exhibited significantly greater gains in knowledge and attitude scores, aligning with cognitive-developmental theory, which suggests that adolescents’ advanced reasoning abilities enhance comprehension of abstract nutrition concepts and disease-risk linkages^67,68^. However, the relationship between age and practice was weaker, suggesting that as adolescents gain autonomy in food purchasing and social eating, external influences such as peer norms, taste preferences, and exposure to unhealthy foods play important roles in shaping the retention of educational messages^69,70^. Gender differences were also observed, with boys showing greater post-intervention improvements in attitude, practice, and micronutrient-rich food intake compared with girls. In many low- and middle-income contexts, adolescent girls experience restricted autonomy over meal decisions and may have limited access to animal-source or fortified foods despite comparable knowledge gains^71^. Boys, in contrast, may benefit from preferential allocation or greater decision-making autonomy regarding snacks and meals. These dynamics likely influenced the observed consumption patterns, underscoring the need for gender-responsive program design, and ensuring that interventions empower girls through experiential learning, peer support, and family engagement components. Also, we observed session attendance (≥ 80%) to be a predictor across all outcomes, emphasizing that program exposure and continuity are critical determinants of intervention success.

In summary, the key strengths of our study include: (i) the use of the IM approach to develop and implement the education program that allowed an effective mapping of intervention protocol to the hypothesized determinants and desirable outcomes of behavior change. This systematic process of program planning and delivery improved both the internal validity and practical relevance of the intervention with the potential for scalability and integration within existing school health and nutrition programs in India, (ii) a targeted focus on micronutrient literacy including sessions that were designed to improve knowledge, understanding, and application of information related to essential vitamins and minerals—specifically their physiological roles, dietary sources, deficiency symptoms, and strategies for adequate intakes in children. The educational resources and intervention extended beyond generic nutrition awareness to encompass the ability to identify micronutrient-rich foods, understand how preparation and food combinations influence nutrient absorption, and recognize the long-term health consequences of deficiencies (iii) an evaluation of intervention across multiple KAP domains (knowledge, attitudes and both healthy and unhealthy dietary practices), ensuring a richer assessment of intervention effectiveness; and (iv) the selection of government schools at study sites to reach children belonging to **l**ow socio-economic backgrounds. The majority of nutrition education studies in India have been conducted in private schools, where students typically have better access to information, food diversity, and family support systems. Our intervention was designed to empower children to make choices aligned with nutritional adequacy even in resource-constrained settings.

The findings of this study have pertinent relevance for ongoing national initiatives such as Poshan Abhiyaan and existing school health and nutrition programs aimed at improving dietary behaviors and combating the micronutrient malnutrition in children across the socioeconomic status. The positive results show that within existing school systems, the IM components that align with routine academic curricula and require minimal additional resources are the most scalable. In particular, needs assessments through discussions and interviews with teachers and parents, integration of theory driven lesson plans aligned with the behavior change outcomes into the regular class periods, and delivery of standardized educational modules to children are feasible, as they leverage existing personnel, timetables, and infrastructure. Additionally, the use of low-cost, culturally adapted educational materials help to consolidate classroom learnings and when embedded within existing curricula and supported by simple monitoring tools, represents a practical and sustainable approach for large-scale implementation in school settings.

Several limitations should also be acknowledged. First, dietary practice outcomes were based on self-reported measures rather than objective dietary assessment or biomarkers with a potential for social desirability or recall bias. Second, while the quasi-experimental pre–post design enabled practical implementation within school settings, the absence of randomization and control groups limits causal inference and increases susceptibility to external influences, testing effects, and regression to the mean. Logistical, time, and resource constraints related to integrating the intervention within existing academic curricula, along with administrative requirements to deliver the intervention uniformly to all eligible students within participating schools, informed the selection of a quasi-experimental design for this study. Furthermore, this investigation was designed as a proof-of-concept study to assess the feasibility, acceptability, and preliminary effectiveness of a micronutrient literacy intervention in school settings in India, thereby providing preliminary data and a strong foundation for subsequent controlled evaluations. Third, our study was designed to capture immediate post-intervention changes only due to time and resource constraints. The KAP questionnaire used to determine the impact of the intervention was adapted from existing validated questionnaires and publicly available resources used to address anemia and micronutrient deficiencies in India. Although the questionnaire was reviewed by subject-matter experts to establish content validity and translated to local languages to ensure linguistic accuracy and conceptual equivalence, rigorous psychometric testing, such as assessment of internal consistency and construct validity, was not conducted. Finally, given that the intervention was conducted in government schools in Mumbai, the generalizability to rural or private school settings should also be interpreted with caution.

## Conclusions

This study provides robust evidence that systematically designed, school-based nutrition education can enhance micronutrient literacy and promote healthier eating behaviors among children and adolescents. The Intervention Mapping framework strengthened program effectiveness and behavioral outcome mapping, contributing to measurable improvements across KAP domains. To translate these promising proximal gains into longer-term improvements in micronutrient status and health, future research should consider longitudinal and controlled designs with longer follow-up intervals and inclusion of objective outcomes such as dietary biomarkers, dietary diversity indices, and anthropometric measures. Implementation research should explore scalable models of IM-based interventions that integrate teacher training, digital reinforcement, and parental participation.

Given the persistent high prevalence of micronutrient inadequacies among Indian schoolchildren, implementation strategies that combine nutrition education with practical, environmental strategies (such as school meal modification, fortified foods, point-of-choice labelling, parental engagement) and enable cost-effective scale up within school systems are warranted. Our findings advocate for early and sustained nutrition education, beginning in late primary school and continuing through adolescence. Moreover, the focus on micronutrient literacy aligns directly with national and global nutrition priorities aimed at reducing anemia, vitamin A deficiency, and other forms of hidden hunger among children and adolescents. Integrating this model into school systems could therefore contribute to achieving Sustainable Development Goal 2 (Zero Hunger) and India’s Poshan Abhiyaan objectives by strengthening the behavioral component of school nutrition policies. Such investments will not only strengthen knowledge and self-efficacy but also contribute to long-term public health gains in combating the burden of malnutrition and micronutrient deficiencies among children in India.

## Data Availability

All data generated and /or analyzed during the present study are provided as a part of the manuscript. Additional information is available from the corresponding author upon reasonable request.
